# Applicability of the modified Emergency Department Work Index (mEDWIN) at a Dutch emergency department

**DOI:** 10.1371/journal.pone.0173387

**Published:** 2017-03-10

**Authors:** Steffie H. A. Brouns, Klara C. H. van der Schuit, Patricia M. Stassen, Suze L. E. Lambooij, Jeanne Dieleman, Irene T. P. Vanderfeesten, Harm R. Haak

**Affiliations:** 1 Department of Internal Medicine, Máxima Medical Centre, Eindhoven/Veldhoven, the Netherlands; 2 Department of Internal Medicine, division of general medicine, section acute medicine, Maastricht University Medical Centre, Maastricht, the Netherlands; 3 Máxima Medical Centre Academy, Máxima Medical Centre, Eindhoven/Veldhoven, the Netherlands; 4 School of Industrial Engineering, Eindhoven University of Technology, Eindhoven, the Netherlands; 5 Maastricht University, Department of Health Services Research, and CAPHRI School for Public Health and Primary Care, Maastricht, the Netherlands; Yokohama City University, JAPAN

## Abstract

**Background:**

Emergency department (ED) crowding leads to prolonged emergency department length of stay (ED-LOS) and adverse patient outcomes. No uniform definition of ED crowding exists. Several scores have been developed to quantify ED crowding; the best known is the Emergency Department Work Index (EDWIN). Research on the EDWIN is often applied to limited settings and conducted over a short period of time.

**Objectives:**

To explore whether the EDWIN as a measure can track occupancy at a Dutch ED over the course of one year and to identify fluctuations in ED occupancy per hour, day, and month. Secondary objective is to investigate the discriminatory value of the EDWIN in detecting crowding, as compared with the occupancy rate and prolonged ED-LOS.

**Methods:**

A retrospective cohort study of all ED visits during the period from September 2010 to August 2011 was performed in one hospital in the Netherlands. The EDWIN incorporates the number of patients per triage level, physicians, treatment beds and admitted patients to quantify ED crowding. The EDWIN was adjusted to emergency care in the Netherlands: modified EDWIN (mEDWIN). ED crowding was defined as the 75^th^ percentile of mEDWIN per hour, which was ≥0.28.

**Results:**

In total, 28,220 ED visits were included in the analysis. The median mEDWIN per hour was 0.15 (Interquartile range (IQR) 0.05–0.28); median mEDWIN per patient was 0.25 (IQR 0.15–0.39). The EDWIN was higher on Wednesday (0.16) than on other days (0.14–0.16, p<0.001), and a peak in both mEDWIN (0.30–0.33) and ED crowding (52.9–63.4%) was found between 13:00–18:00 h. A comparison of the mEDWIN with the occupancy rate revealed an area under the curve (AUC) of 0.86 (95%CI 0.85–0.87). The AUC of mEDWIN compared with a prolonged ED-LOS (≥4 hours) was 0.50 (95%CI 0.40–0.60).

**Conclusion:**

The mEDWIN was applicable at a Dutch ED. The mEDWIN was able to identify fluctuations in ED occupancy. In addition, the mEDWIN had high discriminatory power for identification of a busy ED, when compared with the occupancy rate.

## Background

Emergency department (ED) crowding is a well-known logistical problem affecting emergency care worldwide [1]. ED crowding occurs when the need for emergency services exceeds available resources at the ED or in the hospital [2]. It is associated with adverse patient outcomes, reduces the probability of receiving high-quality care [3–6] and leads to prolonged emergency department length of stay (ED-LOS), delays in treatment and preventable medical errors [7–11]. In addition, crowding at the ED results in ambulance diversion and patients leaving the ED without being seen by a physician [3,9,12].

ED crowding seems to be less evident in the Netherlands [1,13,14]. ED boarding, defined as the inability to transfer admitted ED patients to hospital beds, ambulance diversion and patients leaving the ED without being seen are rare events [14,15]. Nonetheless, 68% of ED managers participating in a Dutch survey study reported that ED crowding, defined as having more patients in the ED than treatment rooms or more patients than staff should ideally care for, occurred at least twice a week [13].The anticipated changes in the organization of emergency care in the Netherlands, involving potential closure of EDs as a cost reduction measure, will impact the availability of emergency services and possibly contribute to ED crowding in the future [16]. To monitor the impact on EDs occupancy and allow timely control measures, an adequate measure of ED occupancy and ED crowding is needed.

The extent of ED crowding is difficult to estimate, as there is no general definition or a gold standard other than physician perception of ED crowding [17]. To better understand and manage crowding, and to compare crowding levels across hospitals, several quantitative and objective ED crowding measures have been developed, such as the Emergency Department Work Index (EDWIN), the National ED Overcrowding Scale (NEDOCS), and the occupancy rate [18–20]. The EDWIN, the NEDOCS and the occupancy rate have all been developed and validated in the USA with an emergency care system characterized by high numbers of ED visits and ED boarding [1]. Both EDWIN and NEDOCS are highly associated with physicians’ perception of ED crowding [21]. However, the NEDOCS quantifies ED crowding based on the number of respirators at the ED, longest admission time, and waiting room time of the last patient [20], which requires more detail than routinely stored in electronic hospital records. The occupancy rate is based on the ratio of the total number of ED patients to the total number of licensed treatment beds per hour. However, urgency level, an important factor influencing workload of ED personnel, is not taken into consideration. Furthermore, studies concerning crowding measures have mostly been applied to settings in the USA, Canada and Australia, and were conducted over a short period of time [21].

The primary objective of the present study was to explore the value of the EDWIN as a measure to track occupancy at an ED in the Netherlands and its ability to identify fluctuations in ED occupancy per hour, day, and month. Our secondary objective was to investigate the discriminatory value of the EDWIN in detecting crowding, as compared with that of the occupancy rate and prolonged ED-LOS.

## Methods

### Study design, setting and participants

The Institutional Review Board of the MMC confirmed that the Medical Research Involving Human Subjects Act (WMO) was not applicable to this study. A retrospective cohort study was performed at Máxima Medical Centre (MMC), a 550-bed teaching hospital in the Netherlands with approximately 28,000 ED visits per year [22]. The ED consists of a triage room and 18 treatment beds divided over 16 treatment rooms, including rooms for trauma, shock and pediatric patients. The primary modes of presentation to the ED are referral by a general practitioner (GP) or self-referral. Other modes of presentation are referral by medical specialists or ambulance [14]. Triage at presentation is routinely performed using the Manchester Triage System (MTS). This five-level system categorizes ED patients into one of the following urgency levels: 1. red (requires immediate assessment), 2. orange (very urgent, requires evaluation within 10 minutes), 3. yellow (urgent, requires evaluation within one hour), 4. green (standard, requires evaluation within 2 hours), and 5. blue (non-urgent, requires evaluation within 4 hours) [23]. The least urgent MTS category (i.e. blue) is not used at our ED, as this uncomplicated patient group would be assessed by a general practitioner. A medical student, a non-trainee (physicians who have not yet started traineeship in a clinical specialty) or trainee resident, or an emergency physician will assess the patients presenting to the ED, supervised by a medical specialist. Health insurance is available for every citizen in the Netherlands. Health care costs of uninsured individuals are covered by the Dutch state.

Data on all ED visits between September 1^st^ 2010 and September 1^st^ 2011 for every medical specialty were extracted from electronic hospital records (ChipSoft EZIS). Given the retrospective observational design and population size, no informed consent was obtained. To ensure patient privacy, we pseudo-anonymized data after data extraction by replacing all identifying variables for the database with a unique study patient code. A password protected key file was stored on the secure internal server of the MMC and only accessible by the responsible investigator (SB). ED visits were excluded when there was an overt incorrect ED recording time or when the patient was directly transferred to another department.

### Data collection

The following data were retrieved from electronic patient and hospital records: age, gender, MTS triage level, date and time of the ED visit, mode of presentation (referral by GP, medical specialist, ambulance or self-referral), number of diagnostic tests performed at the ED (blood, arterial blood-gas, urine or culture tests, electrocardiogram (ECG), X-ray, ultrasonography, computed tomography (CT) scan or magnetic resonance imaging (MRI)), number of consultations by medical specialties at the ED, and medical procedures performed at the ED (intubation, placement of urinary catheter or gastric tube, cardiac rhythm monitoring, administration of oxygen, and application of bandages or casts). ED recording times (in minutes) were sectioned into: 1. ED arrival time, 2. time spent in the waiting room, calculated as the time from ED arrival to ED bed placement, 3. time when triage started, 4. time when treatment was started, 5. time when treatment ended, 6. ED-LOS calculated as the time between ED arrival and the end of treatment with subsequent ED discharge or hospital admission. Information on final disposition, including patients who left the ED without being seen by a physician and date of admission and discharge were retrieved.

### Endpoints

The primary endpoint of the study was the ability of the EDWIN to track occupancy. The secondary endpoint was the ability of the EDWIN to identify ED crowding as compared to the occupancy rate and ED-LOS.

The EDWIN was calculated using the following formula [18]: EDWIN = ∑ n_i_t_i_ / N_a_ (B_T_—B_A_), where n_i_ = the number of patients present at the ED, including the patients in the waiting room, triage and in ED beds, per triage level i; t_i_ = the triage category (scale 1–5, 5 being most acute); N_a_ = number of attending physicians at the ED; B_T_ = the total number of staffed beds at ED; B_A_ = the number of admitted patients (i.e. boarding patients: ED patients already admitted but who are not able to be transferred to hospital beds) in the ED [18].

The EDWIN was adjusted to emergency care in the Netherlands: modified EDWIN (mEDWIN). Four adjustments to the scale of the EDWIN were necessary to make it applicable to our ED [18]. First, as the MTS, a five-level triage system, is used in our ED, we used this system in the mEDWIN instead of the Emergency Severity Index. As only four MTS categories are being used at our ED, corresponding to the following categories in the mEDWIN: red as category 5, orange as category 4, yellow as category 3, and green as category 2. A missing triage level was coded as 1 and was included in the mEDWIN. Second, since ED boarding does not occur at our ED, B_A_ was converted into occupied treatment beds by any patient, instead of only boarding patients. Third, the mEDWIN could produce mathematical errors under extreme circumstances: if all treatment beds are occupied (B_T_−B_A_ = 0), the denominator of the mEDWIN would become zero (N_a_ x 0 = 0). Therefore, a constant was used for B_T_ to prevent a negative mEDWIN. As there were a maximum of 29 patients occupying ED treatment beds in one hour during the study period, the constant was set on 30. Fourth, we also included residents in N_a_, as ED patients are primarily assessed by residents, who supervised by a medical specialist. The mEDWIN was calculated for each hour of the day, referred to as hour slots. For each patient, the mEDWIN was calculated based on the hour of arrival at the ED, as we postulated that the mEDWIN at the start of the ED visit would have the highest influence on the subsequent ED processes.

The predefined cut-off values of the EDWIN based on research by Bernstein, mostly in the USA, will not apply to our ED (i.e., manageable but active ED = EDWIN <1.5, busy ED = EDWIN: 1.5–2.0, and crowded ED = EDWIN >2.0) [18], due to a much lower volume of patients presenting to our ED and to the alterations made to the mEDWIN. We expected to detect a lower mEDWIN at our ED compared with the international cut-off values. For the purpose of the present study and in absence of validated absolute cut-off value, we defined periods of relative crowding based on the 75^th^ percentile of the hourly mEDWIN scores representing the scores of the busiest hours at our ED. The mEDWIN ≥75^th^ percentile of all calculated mEDWIN scores per hour in our study population was ≥0.28, which was used as a preliminary cut-off for crowding in the analysis.

The occupancy rate was calculated per hour using the following formula [19]:

Occupancy rate = (total number of patients at the ED / total number of treatment beds), where the numerator includes every patient in the ED regardless of location (including waiting room or hallway) and the denominator includes only the licensed treatment beds. Based on the threshold previously proposed by Beniuk et al., an occupancy rate >1 was considered as ED crowding [24].

ED-LOS was calculated in minutes per patient. The cut-off value used to define ED crowding was ED-LOS ≥ 4 hours [25]. A patient having left the ED without being seen by a physician was defined as leaving the ED during the time period starting with the initial registration and ending with the end of treatment [15].

### Data analysis

Data analysis was performed using SPSS, version 21.0 (IBM SPSS Statistics for Windows, Armonk, New York). Arrival times were rounded to the whole hour in order to calculate the mEDWIN. The number of attending physicians (either residents or medical specialists) was derived from the rotation schedule, provided by the ED. As previously mentioned, the number of beds available throughout the entire study period was 30. The number of occupied beds per hour was derived from the number of patients who were treated/assessed in that hour. If no patients were present at the ED, both the occupancy rate and the mEDWIN were set to zero.

Extreme values of ED recording times were checked manually. Missing ED recording times were verified by using ED patient records and completed where possible by manually checking the ED patient records. If the time of the start of treatment was missing, it was calculated by adding the average time in waiting room to the average arrival time.

Descriptive analyses were used to describe the mEDWIN, the occupancy rate (= total number of patients at the ED / total number of treatment beds) and ED-LOS. Normality was checked with histograms and the Kolmogorov-Smirnov test of normality. Continuous variables with a non-normal distribution were tested using the Mann–Whitney U test or the Kruskal–Wallis test, depending on the number of groups. For continuous variables with a normal distribution, T-test and ANOVA (analysis of variance) were used. Variables were described by means with standard deviation (SD) and medians with interquartile ranges (IQR). Comparison of the occurrence of ED crowding (i.e. mEDWIN ≥0.28) between groups was tested using the Chi-square test for categorical variables. The correlation between the mEDWIN, and the occupancy rate, or ED-LOS, was presented as Spearman correlation coefficients for which 95% confidence intervals (CI) were calculated by bootstrapping. Receiver operating (ROC) curves of the mEDWIN versus ED crowding based on an occupancy rate >1, or an ED-LOS ≥4 hours were created and the area under the curve (AUC) was calculated. To describe the ED crowding detection properties of mEDWIN, we calculated the sensitivity and specificity of the mEDWIN compared to the occupancy rate. A two-sided p-value < 0.05 was considered statistically significant.

To determine the effect of missing triage levels on the mEDWIN, a sensitivity analysis was performed by excluding the patients with missing triage levels from the analysis to calculate the median EDWIN, and IQR.

## Results

### Emergency department visits

**Fig 1:**
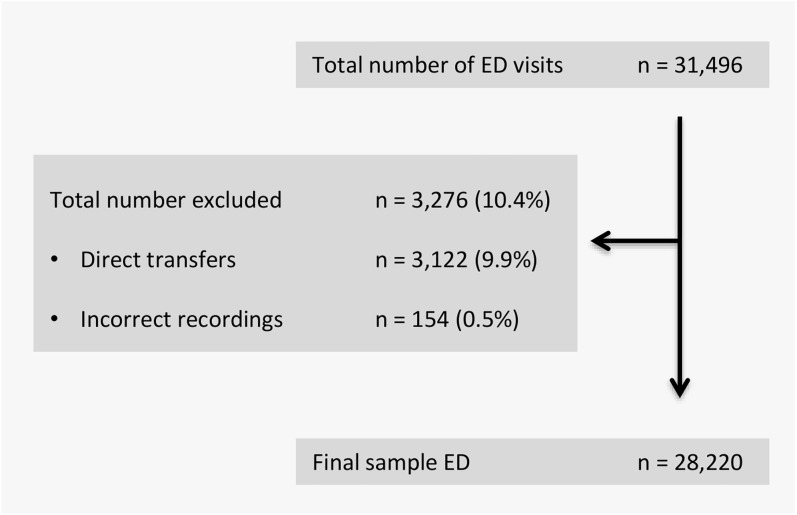
Patient flow diagram. ED = Emergency Department.

During the study period, 31,496 ED visits were recorded in the ED registration system (Fig 1). We excluded 3,122 ED visits by patients (9.9%) who were directly transferred to another department, mostly the cardiac care unit (n = 3,009). Data for 154 ED visits (0.5%) were excluded, due to incorrect ED recording times caused by a computer system malfunction. In total, 28,220 ED visits were included in the analysis, which represented 8,712 hours over 363 days. The median number of physicians or residents at the ED was 9.5 (range 3–10.5) during the hours from 8:00–17:59 h, 8.5 (range 3–9.5) during the hours from 18:00–23:59 h and 4 (range 4–5) during the hours from 0:00–7:59 h.

### Patient characteristics

The mean age of the ED patients was 43.3 years, and 53.7% were male (Table 1). The majority of ED visits were self-referrals (45.9%), followed by referrals by a GP (35.1%). Overall, the patients predominantly presented with urgency levels of green (56.1%) and yellow (31.7%), whereas 0.6% of the patients were classified as red, and 9.7% as orange. The triage level was missing in 551 ED visits (2.0%) and coded as 1. A higher triage level was associated with a higher number of diagnostic tests and medical procedures performed on the ED (both p <0.001) (S1 Table).

**Table 1:** Characteristics of Emergency Department patients.

Characteristic	Total no. ED visits N = 28,220
Mean (SD) age, years	43.3 (26.3)
Male participants (%)	15,141 (53.7)
Day of presentation • Weekday (%) • Weekend (%)	19,858 (70.4) 8,362 (29.6)
Time of presentation • 8.00–16.59 (%) • 17.00–23.59 (%) • 0.00–7.59 (%)	16,344 (57.9) 8,821 (31.3) 3,055 (10.8)
Mode of referral • General practitioner (%) • Ambulance (%) • Medical specialist (%) • Self-referral (%) • Other (%) • Unknown	
	9,898 (35.1)
	2,158 (7.6)
	2,104 (7.5)
	12,954 (45.9)
	1,105 (3.9)
	1 (0.0)
Median time in waiting room in minutes (IQR)	6 (2–22)
Median treatment time minutes (IQR)	77 (39–132)
Disposition • Leave without being seen (%) • Discharge home without follow-up (%) • Discharge home with follow-up (GP/outpatient clinic) (%) • Admission to AMAU (%) • Admission to a hospital ward (%) • Mortuary (%) • Other (nursing home and function department) (%)	
	48 (0.2)
	7,753 (27.5)
	11,869 (42.1)
	6,155 (21.8)
	2,335 (8.3)
	30 (0.1)
	30 (0.1)

SD = standard deviation; ED = emergency department; GP = general practitioner; IQR = Interquartile range; AMAU = Acute Medical Admission Unit.

In 511 cases (1.8%), the timing of the start of treatment was unknown. The median treatment time was 77 minutes (IQR 39–132) (Table 1). In total, 48 patients (0.2%) left the ED without being seen by a physician. In total 8,485 (30.1%) ED visits resulted in admission to the hospital, either to the acute medical admission unit (AMAU) or to a general ward.

### mEDWIN per hour

**Fig 2:**
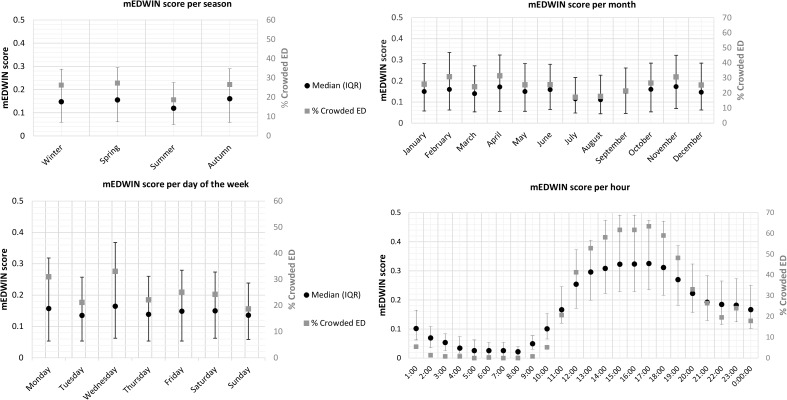
Median mEDWIN with corresponding interquartile range and percentage of ED crowding based on 75^th^ percentile of the mEDWIN per season, month, day and hour. ED = Emergency Department; IQR = Interquartile range.

The median mEDWIN per hour was 0.15 (IQR 0.05–0.28) (Table 2). The distribution of the mEDWIN and percentage of ED crowding (i.e. mEDWIN ≥0.28) differed significantly per hour, day, month and season (Fig 2). Overall, the median mEDWIN and the percentage of ED crowding were higher on weekdays as compared to weekends (0.15 vs. 0.14, p = 0.021, respectively 26.6% vs. 21.5%, p<0.001). The mEDWIN was highest on Wednesday (median 0.16, IQR 0.05–0.37) (Fig 2). Similarly, the occurrence of ED crowding differed among the days of the week, with the highest frequency on Wednesday (p<0.001). During the day, there was a peak in median mEDWIN (0.30–0.33, IQR 0.20–0.49) and ED crowding (52.9–63.4%) between 13:00 and 18:00h (Fig 2), whereas, the median mEDWIN was lowest between 02:00–09:00h (0.05–0.07, IQR 0–0.11). The median mEDWIN was also lower during the summer than in other seasons (p<0.001). Accordingly, ED crowding was less frequent in the summer than in other seasons (18.3% versus 26.3–27.3%, p<0.001).

**Table 2:** Modified Emergency Department Work Index (mEDWIN), occupancy rate and Emergency Department Length of Stay (ED-LOS).

	N	Median	IQR	Range
mEDWIN per hour slot*	8,712	0.15	0.05–0.28	0–9.16
mEDWIN per patient	28,220	0.25	0.15–0.39	0–9.15
Occupancy rate per hour slot	8,712	0.50	0.17–0.83	0–1.89
ED-LOS in minutes per patient	28,220	100	61–152	0–613

N = number; IQR = Interquartile Range

*N = the number of hour slots during the 363 days of the study period.

Based on predefined thresholds of the EDWIN of 1.5–2.0 [18], a busy ED occurred in 0.1% of the hour slots in our study. Furthermore, in only 0.1% of the hour slots, the ED was classified as crowded based on the thresholds by Bernstein (i.e. EDWIN >2.0).

### mEDWIN per patient visit

The median mEDWIN for all patient visits was 0.25 (IQR 0.15–0.39) (Table 2). Patients <65 years (73.4%) presented at moments with a lower median mEDWIN (0.25, IQR 0.15–0.38) than patients ≥65 years (26.6%) (0.26, IQR 0.16–0.40, p<0.001). In patients who were hospitalized following the ED visit (30.1%), the median mEDWIN at the ED upon first presentation was similar to that of patients who were discharged from the ED (median 0.25, IQR 0.15–0.38 vs. 0.25, IQR 0.15–0.39, respectively, p = 0.851). The median mEDWIN was 0.32 (IQR 0.24–0.46; 62.5% during ED crowding) during the visits of the 48 patients who left the ED without being seen versus 0.25 (IQR 0.15–0.39; 42.2% during ED crowding) for the visits in which patients had been seen by a physician(p = 0.005).

Excluding visits with missing triage levels did not change the median mEDWIN (0.25, IQR 0.15–0.39).

### Comparison of mEDWIN with occupancy rate and ED-LOS

The median occupancy rate was 0.50 (IQR 0.17–0.83) (Table 2). ED crowding (occupancy rate per hour >1) occurred in 11.5% of ED visits. On Mondays and Fridays, the ED was crowded in 19.2% and 14.9% of visits, respectively (p<0.001 compared with the other days). The occupancy rate mainly exceeded 1 at ED visits between 12:00–19:00h (20.9–35%). Overall, an occupancy rate >1 occurred less frequently during the summer (6.1%) as compared to other seasons (12.2–13.7%, p<0.001).

The median ED-LOS was 100 minutes (range 0–613, IQR 61–152 minutes) (Table 2). In 5.3% of the patients, ED-LOS exceeded 4 hours. These patients mostly presented on Monday (6.8%), Tuesday (6.4%), and Friday (7.0%). An ED-LOS ≥4 hours occurred most often at 7:00, 12:00 and 13:00 (9.4, 7.5 and 7.8%, respectively) and least often between 1:00–4:00h (1.2–2.3%) (p<0.001). As predicted by occupancy rate, an ED-LOS ≥4 hours was least frequent during the summer (3.1%).

**Fig 3:**
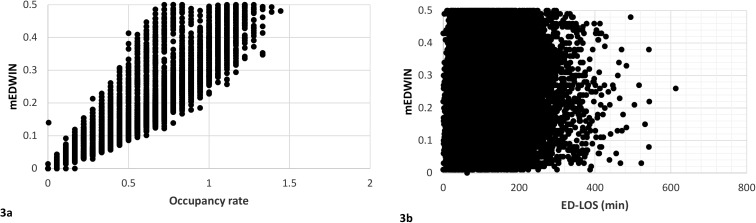
**Comparison of results for the modified Emergency Department Work Index (mEDWIN) versus occupancy rate (3a) and Emergency Department Length of Stay (ED-LOS) (3b). 3a.** Spearman correlation coefficient 0.947 (95% CI 0.945–0.950). **3b.** Spearman correlation coefficient 0.16 (95% CI 0.15–0.18).

**Fig 4:**
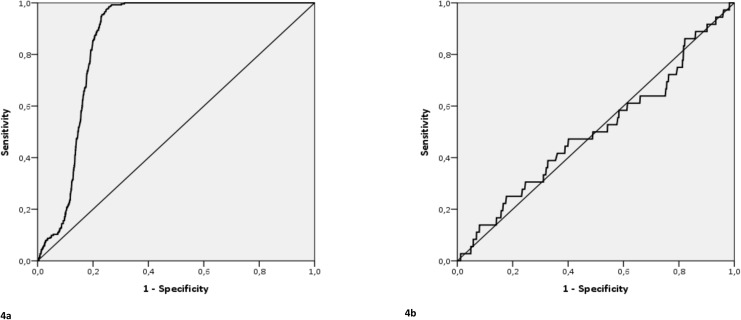
**Receiver operating characteristic curves of the modified Emergency Department Work Index (mEDWIN) for emergency department crowding based on the occupancy rate (4a), and on the Emergency Department Length of Stay (ED-LOS) (4b). 4a.** Crowded ED, i.e. occupancy rate > 1; AUC mEDWIN = 0.86. **4b.** Crowded ED, i.e. ED-LOS ≥ 4 hours; AUC mEDWIN = 0.50.

The Spearman correlation coefficient between the occupancy rate and the mEDWIN was 0.95 (95% CI 0.945–0.950) (Fig 3) and 0.16 (95% CI 0.15–0.18) for mEDWIN and ED-LOS (Table 3). In 13.5% of hour slots with crowding (based on mEDWIN ≥0.28), the occupancy rate was <1. The AUC was 0.86 (95% CI 0.85–0.87) when mEDWIN was compared with an occupancy rate >1 and 0.50 (95% CI 0.40–0.60) when compared with an ED-LOS ≥4 hours (Fig 4).

**Table 3:** Characteristics of the modified Emergency Department Work Index (mEDWIN) versus occupancy rate and Emergency Department Length of Stay (ED-LOS).

	N	Correlation (95% CI)	AUC (95% CI)
mEDWIN versus Occupancy rate	8,712	0.947 (0.945–0.950)	0.86 (0.85–0.87)
mEDWIN versus ED-LOS	28,220	0.16 (0.15–0.18)	0.50 (0.40–0.61)

mEDWIN = modified Emergency Department Work Index; ED-LOS = Emergency Department Length of Stay; AUC = Area Under the Curve

The sensitivity and specificity of the mEDWIN cut-off of 0.28 was 99.5% and 84.5%, respectively.

## Discussion

Our aim was to explore the applicability of the EDWIN to monitor ED occupancy at a Dutch ED. Given the distinct organization of emergency care in the Netherlands, we adjusted the EDWIN to account for both the absence of a boarding system, which is part of the original EDWIN and the use of a different triage system. The modified EDWIN (mEDWIN) was able to identify fluctuations in ED occupancy over a period of time. By using the 75^th^ percentile of mEDWIN as a threshold of ED crowding, we could identify fluctuations in patient flow and periods of relative ED crowding. In addition, the mEDWIN had additional value for identifying ED crowding when compared with the occupancy rate.

Several adjustments to the mEDWIN were made to match the conditions of the acute care] system in the Netherlands. Other studies have applied changes to the formula as well [21,26]. The EDWIN is based on the Emergency Severity Index (ESI), a five-level triage tool associated with resource use and hospitalization rates. In our study, we used the MTS in the mEDWIN. Similar to the ESI, the MTS is highly associated with the number of diagnostic tests and medical procedures performed at the ED, and with the hospital admission rate. The EDWIN was previously evaluated using a non-specified triage system other than the ESI [21]. In addition, another study made adjustments in the calculation of the EDWIN to avoid computational errors by dividing by zero [26]. Furthermore, because the conditions of our ED differ from the hospitals where the EDWIN has been validated, we used altered threshold values to identify a crowded ED. The majority of fluctuations in patient flow and ED crowding would have been unobserved if our results were limited to the predefined cut-off values of the EDWIN determined by Bernstein [18] (i.e., manageable but active ED = EDWIN <1.5, busy ED = EDWIN 1.5–2, and crowded ED = EDWIN >2), as the EDWIN was ≥1.5 in only 0.2% of cases at our ED. This finding is in accordance with another study that found that the accuracy of the EDWIN may be less when ED crowding is less prevalent [21]. Our study reveals that we were able to assess occupancy with the mEDWIN and identify relative busy periods at our ED. However, further research on calibration of the mEDWIN is necessary to find a cut-off which is generalizable to other EDs.

Because the mEDWIN was applied over a period of an entire year, we were able to demonstrate major variations in ED occupancy over seasons and months. A lower mEDWIN and ED crowding during the summer months is understandable, given the decrease in the number of patients visiting the ED during the holiday season. However, this has not previously been quantified. The increase in ED crowding from 13:00–18.00h is consistent with other studies [13,19]. A remarkable finding concerning patient flow was a high mEDWIN and percentage of ED crowding on Wednesdays. This might be explained by a lower number of physicians present in the ED from 8:00–17:00 h on Wednesdays compared with other days, resulting in a smaller numerator, and thus a higher mEDWIN.

A general definition of ED crowding and a gold standard to quantify ED crowding is still lacking, although several ED crowding measures have been developed [18–20]. Our findings show a strong correlation between the mEDWIN and the occupancy rate. When using an occupancy rate >1 as cut-off for ED crowding, the mEDWIN has adequate discriminatory value (AUC 0.86), which is in accordance with other studies [8,19]. Sensitivity and specificity to detect ED crowding as compared with the occupancy rate was 99.5% and 84.5% respectively. The variables of the formula of the occupancy rate are included in the mEDWIN as well, which explains the strong correlation. Other research has suggested the superiority of the relative simple occupancy rate compared with other crowding measures, such as EDWIN or NEDOCS [8,19,26–28]. However, the mEDWIN may have added value compared with the occupancy rate, as periods of relative crowding were frequently (13.5%) observed based on the mEDWIN (i.e., ≥0.28), while the occupancy rate remained <1.0. The main advantage of the EDWIN compared with the occupancy rate is the incorporation of the triage level in the score for quantifying ED crowding, as one critically ill patient may influence ED crowding more than several patients with minor injuries. Only 0.1% of hour slots with ED crowding based on the occupancy rate (i.e. >1) was missed with the mEDWIN.

In contrast, the correlation between the mEDWIN and the ED-LOS was weak. For instance, the AUC of the mEDWIN compared with an ED-LOS ≥4 hours was only 0.50. ED-LOS has previously been marked as an objective measure to assess ED crowding with great reproducibility [17]. However, additional factors may contribute to longer ED-LOS, such as consultations by different specialties and the experience of the physician, which may only influence ED-LOS in a small number of patients and not ED crowding in general [29,30]. Furthermore, patients with a high urgency level can create high workload, resulting in a high mEDWIN, even when their ED-LOS may be short.

The most commonly used model of ED crowding is the input-throughput-output model [3,17,31]. However, in contrast to emergency care in the USA, Canada and Australia, ED crowding in the Netherlands is primarily based on input and throughput factors rather than output factors, which reflect problems associated with the disposition of ED patients [3,17,31]. This is consistent with other countries with a comparable primary care system, such as Scandinavian countries [1]. The most important input and throughput factors are directly or indirectly included in the mEDWIN: the number of patients in the ED, their acuity level and resource use, the number of physicians at the ED and the number of occupied beds. Our results suggest that the mEDWIN can serve as an adequate measure for monitoring ED occupancy by including organizational factors as well as urgency level, where ED crowding based on previously defined values is infrequent or not taking into account severity and urgency levels.

### Limitations

Our results may have been influenced by several limitations. Firstly, there is a risk of bias due to the retrospective observational design. It is possible that data such as ED recording times are incomplete or incorrect. However, the effect of missing values of the triage level (2.0%) on the mEDWIN was minimal as shown in a sensitivity analyses. Some misclassification of the mEDWIN assigned to patients may have occurred, because we rounded the arrival times of patients to the whole hour. Secondly, because of the single-center setting, our findings may be less generalizable. Thirdly, the EDWIN was modified to better suit the Dutch emergency care system (mEDWIN), since ED crowding and ED boarding are infrequent. Although the health care system is well organized in the Netherlands, this distinct organization should be taken into account in interpreting our findings. In addition, we applied a different classification of ED crowding based on the observed mEDWIN. ED crowding was based on the upper quartile of the mEDWIN per hour (i.e., ≥0.28). Although the relevance of a mEDWIN ≥0.28 is uncertain, it is useful for the identification of the busiest periods at the ED, in particular because no standard method for measuring ED crowding exists. Nonetheless, our results may be applicable to sites with a similar organization of primary care, and where ED crowding occurs less frequently as well. Lastly, the influence of medical students or ED nurses on ED crowding was unclear and therefore not considered in this study.

### Future perspectives

Emergency care in the Netherlands is on the verge of major changes, involving possible mandatory closure of EDs and a more prominent role for general practitioners. Consequently, the case mix of ED patients is expected to change, as higher numbers of less complex patients are expected to visit general practice centers after hours, and more complex patients will present to the ED. Since the number of ED visits is not expected to decrease proportionately, ED crowding will increase accordingly. More research is necessary to be able to monitor future trends and to anticipate and adapt to the altered patient flow. This study was the first to apply the EDWIN at an ED in the Netherlands.

Future prospective studies may focus on the identification of threshold values of the mEDWIN in an emergency care system where ED crowding occurs less frequently. In addition, comparison of the mEDWIN with physician and patient perception of ED crowding may add valuable information. Moreover, the development of an ED simulation model, incorporating the mEDWIN, could be beneficial in predicting ED crowding and implementing strategies to better manage crowding, for example scheduling more ED personnel from 13:00–18:00 h. In addition, the burden of different medical specialties on ED crowding, such as surgical or medical patients, might be evaluated separately. Further studies on the influence of ED crowding on patient outcome through the use of quantitative measures, such as the mEDWIN, are needed as well.

## Conclusion

After minor adjustments, the EDWIN (mEDWIN) was applicable as a monitoring tool for ED occupancy and relative crowding at our ED. The mEDWIN was able to identify fluctuations in ED crowding per hour, day and month. In addition, the mEDWIN demonstrated high discriminatory power for the identification of relative ED crowding, as compared with the occupancy rate. ED-LOS was not an appropriate measure to predict ED crowding. Our findings suggest that the mEDWIN can serve as a valid measure for detecting ED crowding at a Dutch ED. Further prospective research is necessary to validate threshold mEDWIN values.

## Supporting information

S1 Table Number of diagnostic tests and medical procedures performed at the emergency department per urgency level. (DOCX)
