# Supplementary material for: Applicability of the modified Emergency Department Work Index (mEDWIN) at a Dutch emergency department
Source: PLoS One. 2017 Mar 10;12(3):e0173387. doi: 10.1371/journal.pone.0173387 (PMC5345800; doi:10.1371/journal.pone.0173387)
Supplement: S1 Table — (DOCX) [file pone.0173387.s001.docx]

**Supporting Information**

**S1 Table. Number of diagnostic tests and medical procedures performed at the emergency department per urgency level.**

| **Urgency level by MTS** | **No. of patients (%)** | **No. of diagnostic tests*** | | | | **No. of medical procedures*** | | | |
| --- | --- | --- | --- | --- | --- | --- | --- | --- | --- |
|  |  | **Median** | **IQR** | **Mean** | **SD** | **Median** | **IQR** | **Mean** | **SD** |
| Red | 158 (0.6) | 3.5 | 2-5 | 3.4 | 2.1 | 4.0 | 3-5 | 3.7 | 1.6 |
| Orange | 2,745 (9.7) | 2.0 | 1-4 | 2.6 | 1.9 | 2.0 | 1-3 | 1.8 | 1.3 |
| Yellow | 8,933 (31.7) | 1.0 | 1-3 | 1.8 | 1.5 | 1.0 | 0-2 | 1.1 | 1.0 |
| Green | 15,833 (56.1) | 1.0 | 0-1 | 0.8 | 1.0 | 1.0 | 0-1 | 0.7 | 0.8 |
| Missing | 551 (2.0) | 0 | 0-0 | 0.4 | 1.1 | 0 | 0-1 | 0.5 | 0.9 |

MTS = Manchester Triage System; IQR = Interquartile range; SD = standard deviation; * = p < 0.001.
